# Poroma: A case report of pulsatile papule visualized on dermoscopy

**DOI:** 10.1002/ccr3.2520

**Published:** 2019-11-05

**Authors:** Thanadon Eksomtramage, Kumpol Aiempanakit

**Affiliations:** ^1^ Division of Dermatology Department of Internal Medicine Faculty of Medicine Prince of Songkla University Hat Yai Thailand

**Keywords:** adnexal neoplasm, blinking light, dermoscopy, poroma, pulsatile

## Abstract

Poroma, a benign sweat tumor, commonly presents with a nontender papule on the extremities. It can appear with a blinking light appearance on dermoscopy in real time.

## Graphical abstract

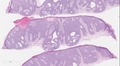

## 1 BACKGROUND

We report the case of an Asian female patient who presented with a pink papule on her left index finger for a year. Dermoscopic findings showed the characteristics of poroma. In addition, we found the blinking light appearance of the pulsatile vascular structure in real time.

Poroma is an uncommon benign cutaneous adnexal neoplasm that proceeds from an intraepidermal sweat gland lineage and acrosyringium. The pathogenesis of the tumor relates to scarring, radiation, and trauma, after which it slowly grows down to the dermis, connecting with the fibrovascular stroma.

Poroma commonly presents with a nonpainful lesion in adults, which often appears on the fingers, palms, soles, or less frequently, at other sites of the body. It is characterized by a single, well‐demarcated pinkish to reddish color as it progresses from being a papule to a plaque, with a firm nodule consisting of different surfaces (ulcerated, smoothed, and verrucous). Dermoscopy, a noninvasive diagnostic tool, can provide correlated information for differentiating between benign and malignant neoplasms. Several published reports have described the general dermoscopic findings of the poroma as follows: white interlacing areas around vessels, yellow structureless areas, milky red globules, and poorly visualized vessels.^1^, ^2^, ^3^ Herein, we report a case of poroma with a peculiar pulsatile red papule (like off and on) on dermoscopy, presenting an atypical appearance.

## 2 CASE PRESENTATION

**Figure 1:**
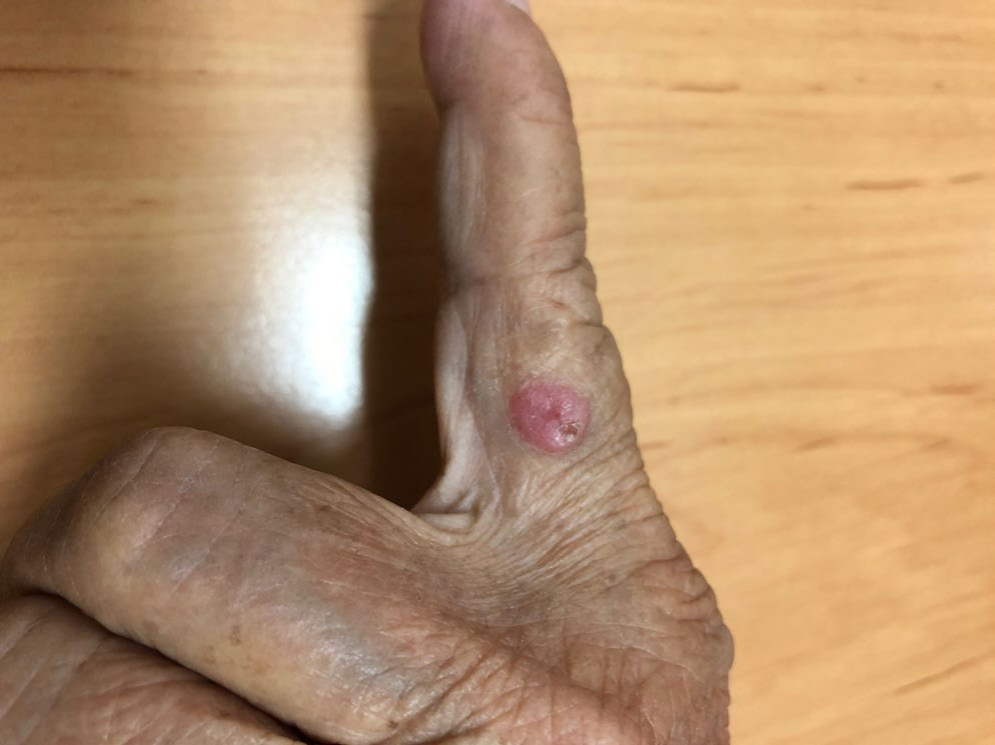
Clinical photograph of the poroma showing a nonpigmented, red papule on the left index finger

An Asian woman in her seventies visited the Dermatology Clinic, Prince of Songkla University, presenting with a slowly progressively enlarging papule on her left index finger for 1 year. Physical examination revealed a single, painless, and shiny erythematous papule with crust and an approximately 0.7‐cm diameter on the ulnar side of her left index finger (Figure 1).

From both her medical history and physical examination, the clinical differential diagnoses included pyogenic granuloma, basal cell carcinoma, squamous cell carcinoma, amelanocytic melanoma, clear cell acanthoma, and keratoacanthoma.

**Figure 2:**
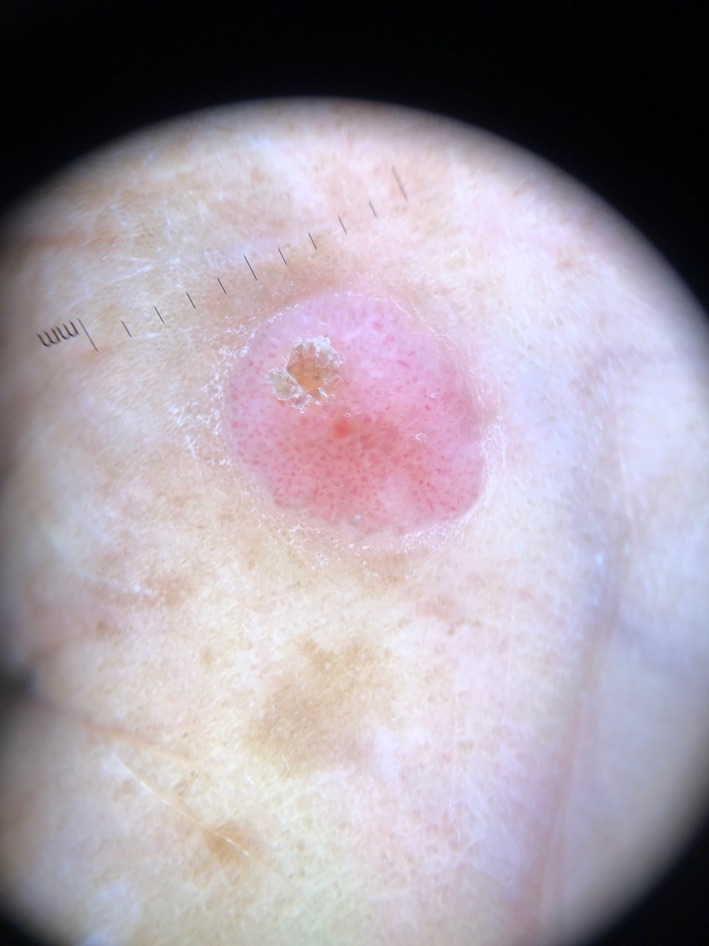
Dermoscopic features of the poroma, showing a chalice shape, “cherry blossom” vessels, a structureless pink‐white area, and hairpin vessels

In this case, we also used immersion polarized dermoscopy to assort to the groups of tumors. We found collarette scaling, a yellow structureless area, polymorphous vessels that included branched vessels with rounded endings (including chalice and flower‐like morphologies), white interlacing areas between vessels, milky red globules, and structureless pink‐white regions (Figure 2). In addition, we detected a blinking light appearance as depicted by the pulsatile vascular structure on a white surface in real time (Video S1).

**Figure 3:**
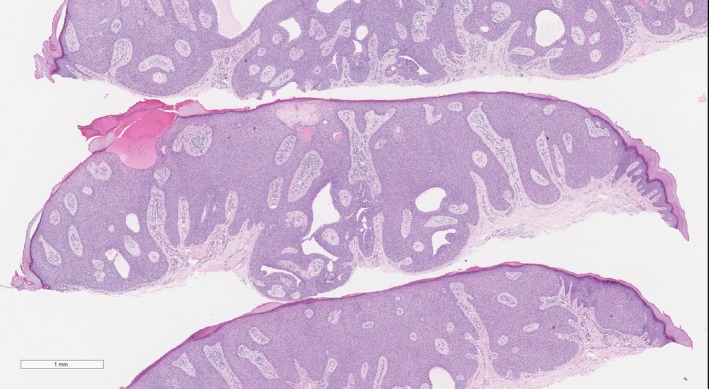
Dermatopathology of the poroma (hematoxylin‐eosin staining, original magnification ×2)

The dermatopathology findings were typical for poroma (Figure 3). All the lesions were formed of cuboidal basophilic cells arranged in anastomosing broad bands, with small ductal spaces in contact with the epidermis and extending onto the dermis. The capillaries were dilated and filled with erythrocytes.

## 3 DISCUSSION

Poroma was first reported in 1956^4^ and represented 10% of all sweat gland tumors. It can occur in both sexes and all age groups; the onset is usually at the age of >40 years.

The International Dermoscopy Society reported 113 poromas. They found, most commonly, nonpigmented papules on the nonacral sites. The dermoscopic findings showed statistically significant poroma with the following features: (a) white interlacing areas around vessels; (b) yellow structureless areas; (c) milky red globules; and (d) poorly visualized vessels. The presence of these findings, with the appearance of branched vessels with rounded endings, has been associated with the disease, at a sensitivity and specificity of 62.8% and 82.0%, respectively.^1^

We report on the unique dermoscopic findings of blinking light appearance as depicted by the pulsatile vascular structure on a white surface in real time. These findings correlated with those from dermatopathology studies that showed that the tumor had grown down to the dermis, connecting with the fibrovascular stroma. To our knowledge, these findings have not been reported in previous studies. In the large study conducted by the International Dermoscopy Society,^1^ only dermoscopic images were examined; hence, unlike in our study, which used a video, this appearance was not reported.

## 4 CONCLUSION

Dermoscopy is a noninvasive diagnostic tool. However, real‐time examinations should additionally be evaluated, as some findings, including those from this case with the blinking light appearance, may be observed only in cases of poroma.

## CONFLICT OF INTEREST

All authors declare that there are no conflicting interests.

## AUTHORS' CONTRIBUTIONS

TE: collected the data and wrote, read, and approved the final manuscript. KA: designed and collected the data and wrote, read, and approved the final manuscript.

## ETHICAL APPROVAL

This case report was approved by the Research Ethics Committee, Faculty of Medicine, Prince of Songkla University (REC. 62‐064‐14‐1).

## CONSENT FOR PUBLICATION

The authors have consent for publication.
