# Supplementary material for: Poroma: A case report of pulsatile papule visualized on dermoscopy
Source: Clin Case Rep. 2019 Nov 5;7(12):2417–9. doi: 10.1002/ccr3.2520 (PMC6935676; doi:10.1002/ccr3.2520)
Supplement: Supplementary file 2 [file CCR3-7-2417-s002.docx]

**Video 1.** Dermoscopic finding of blinking-light appearance.
